# Iodoform

**Published:** 1889-10

**Authors:** 


IODOFORM.—Dr. Emory Lanphear, of Kansas City, Mo., says that, after an extensive use of this "antiseptic," he has entirely abandoned it in favor of other agents—agents better in effects and far less disagreeable in practice. He found salicylate of sodium to serve an excellent purpose as a dressing in a purulent pleuritis where resection of a rib had been resorted to, and where alarming toxic symptoms had followed the use of iodoform.

We have found iodoform, that we formerly regarded with great favor, as entirely unsuited to gynecic uses, and have likewise entirely abandoned it.

---

THE BUFFALO *Sunday Express*, in its issue of September 22, 1889, published a most sensible editorial upon Infant Mortality in Buffalo, using as a text therefor the valuable paper upon that subject by Dr. Irving M. Snow, of this city, published in the September number of this journal. The *Express*, however, probably inadvertently, failed to credit the Buffalo MEDICAL AND SURGICAL JOURNAL with the publication of the article.

---

WE PRINT an advertisement in this issue of our journal showing some remarkable statements from the well-known manufacturing and importing druggists, Messrs. Tarrant & Co., New York, which are based upon a recent court decision in Berlin. Every physician who is interested in preventing substitutions, or in obtaining genuine preparations, should give the communication a careful perusal. The time has gone by when business can long thrive through misrepresentation, and we hope for the sake of all concerned that the alleged irregularities may be cleared up or prove unfounded.

---

THE NUMBER OF STUDENTS attending the medical schools of Germany has increased to such an extent in the past few years, that the government has been petitioned to open new schools of medicine. The government refuses to do this, but proposes to remedy the evil by exacting higher tuition fees, and lengthening the course of study required for graduation.

---

IN DRAWING up the rules governing the proposed governmental university for Russia, the following paragraph, relating to the patients treated by the professors of this school, appears: "If a patient dies, the professor will be permitted, no reason or pretext being able to prevent, to make an autopsy of said patient in the presence of the students."

MENTHOL, according to the *Prager Med. Wochenschr.*, acts as a good anodyne, and is valuable in the local treatment of laryngeal tuberculosis. A ten per cent. solution should be used in the beginning, to be gradually increased in strength; a forty to fifty per cent. solution, however, causes severe irritation.

---

THE INTERNATIONAL SCIENTIFIC CONGRESS OF ANTI-VACCINATORS held its fifth annual meeting at Paris, September 3d, 4th, and 5th. The object of the Congress is to enlighten public opinion (?) upon this "grave hygienic question."

---

HYPNOTISM.—The Council-General of the Department "Lower Seine" (France) has adopted a resolution, presented by Drs. Fauvel and Lecompte, interdicting all open exhibitions of hypnotism in that department.

---

THE *Chicago Medical Journal and Examiner* has suspended publication. This is noted with regret, for good medical magazines are not so plentiful as to disappear without being missed.

---

AT THE International Congress of Archeologists and Anthropologists, recently held at Paris, 420 delegates were present, among whom were five Americans.

---

THE GROSS MEDICAL COLLEGE, of Denver, will require a three-years' graded course for graduation after September 1, 1890.

